# Frontiers in premature beats research: a bibliometric analysis

**DOI:** 10.3389/fcvm.2024.1343274

**Published:** 2024-07-12

**Authors:** Dan Wu, Xiaojing Xia

**Affiliations:** ^1^ Department of Internal Medical, Nanchang HongDu Hospital of Traditional Chinese Medicine, Nanchang, Jiangxi, China; ^2^ Affiliated Eye Hospital of Nanchang University, Jiangxi Medical College, Nanchang University, Nanchang, China

**Keywords:** premature beats, arrhythmia, ventricular premature beats, VOSviewer, visualization

## Abstract

**Background:**

This study aimed to assess the scientific results and activities of premature beats research from a global perspective.

**Methods:**

Publications related to premature beats published between 2003 and 2024 were identified and selected from the Web of Science core collection. VOSviewer was used to conduct co-authorship, co-citation, and co-occurrence analyses of the authors, organizations, countries/regions, references, sources, cited authors, and keywords.

**Results:**

In total, 5,283 publications on the topic of premature beats were identified from the Web of Science core collection. The number of publications on this topic has steadily grown since 2003. Fred Morady, Frank Bogun and Krit Jongnarangsin were the top three researchers with the strongest total link strengths. The University of Washington, Johns Hopkins University, and the University of Minnesota are the top three organizations with the strongest total link strengths. The United States has made the greatest contributions to the field of premature beats. Haïssaguerre, M et al.'s publication in The New England Journal of Medicine in 1998 entitled “Spontaneous initiation of atrial fibrillation by ectopic beats originating in the pulmonary veins” is the most cited reference. The most cited references come from the journal named Circulation. Haïssaguerre, M has the highest number of citations. The keywords for all current publications can be divided into four categories: “mortality rate,” “risk and prevention,” “mechanism,” and “classification and treatment.”

**Conclusions:**

This bibliometric study provides insights into the current status and research trends in premature beats over more than 20 years. Future research will focus on an in-depth exploration of the nature of premature beats, especially ventricular premature beats, mastering the development law of premature beats, and optimizing existing detection methods.

## 1 Introduction

Premature beats, also known as ectopic beats or premature contractions, are a common type of cardiac arrhythmias. They refer to heartbeats that occur earlier than expected because of impulses originating from ectopic pacemaker sites, while the underlying heart rhythm remains regular (1). Based on their site of origin, premature beats can be categorized as ventricular, atrial, or junctional (2, 3). With an aging population and widespread use of dynamic electrocardiogram monitoring, the prevalence of premature beats has increased in the general population (4, 5). Premature beats in structurally normal hearts are usually benign; however, in the presence of structural heart disease, they can contribute to various cardiovascular conditions, such as myocardial diseases, heart failure, and sudden cardiac death, becoming a major cause of morbidity and mortality in cardiovascular diseases (6–8).

Since Sir Thomas Lewis proposed “aberrant beats” in 1910 (9), scholars have been interested in the potential pathophysiological processes of ventricular premature beats in clinical and translational research (10–12). Some researchers have focused on the prevention and basic treatment approaches for atrial premature beats in clinical strategy (13–15). Additionally, there has been attention to the classification and significance of junctional premature beats in basic research and clinical practice (3, 16–18). With advancements in medical care, premature beats have become an increasingly important factor in the development of cardiovascular diseases, prompting scholars to conduct in-depth research.

Bibliometric analysis is a quantitative analytical method that explores information within a large body of literature, including publications, authors, organizations, countries/regions, references, sources, cited authors, keywords, *etc*. By utilizing the VOSviewer software (19), a tool for constructing and visualizing bibliometric networks, scholars can uncover the inherent connections between information and further understand the trends and cutting-edge developments in their research field. Despite nearly a century of research on premature beats, a bibliometric visualization analysis has not been conducted. This study aimed to employ VOSviewer software to provide a visual and quantitative description and evaluation of premature beats research from 2003 to May 31,2024. It comprehensively organizes and presents representative information networks and offers a theoretical reference for identifying new research hotspots in the field of premature beats.

## 2 Methods

This article was indexed based on raw data from the Web of Science core collection (WoScc). The keywords for premature beats were obtained from MeSH (https://www.ncbi.nlm.nih.gov/mesh) searches.

Topic#1 = Cardiac Complexes, Premature or Complexes, Premature Cardiac or Extrasystoles or Premature Beats or Beat, Premature or Beats, Premature or Premature Beat or Premature Cardiac Complex or Premature Cardiac Complexes or Cardiac Complex, Premature or Premature Cardiac Complices or Ectopic Heartbeats or Ectopic Heartbeat or Heartbeat, Ectopic or Heartbeats, Ectopic or Extrasystole.

Topic#2 = Ventricular Premature Complexes or Extrasystole, Ventricular or Ventricular Extrasystole or Ventricular Extrasystoles or Premature Ventricular Beats or Premature Ventricular Beat or Ventricular Beat, Premature or Ventricular Beats, Premature or Premature Ventricular Complex or Ventricular Complex, Premature or Premature Ventricular Contractions or Premature Ventricular Contraction or Ventricular Contraction, Premature or Ventricular Contractions, Premature or Ventricular Ectopic Beats or Ectopic Beats, Ventricular or Ectopic Beat, Ventricular or Ventricular Ectopic Beat or Ventricular Premature Complex.

Topic#3 = Atrial Premature Complexes or Premature Complexes, Atrial or Atrial Ectopic Beats or Atrial Ectopic Beat or Ectopic Beat, Atrial or Ectopic Beats, Atrial or Atrial Premature Complex or Atrial Premature Complices or Premature Complex, Atrial or Premature Complices, Atrial or Extrasystole, Atrial or Atrial Extrasystole or Atrial Extrasystoles or Extrasystoles, Atrial or Premature Atrial Beats or Atrial Beat, Premature or Atrial Beats, Premature or Premature Atrial Beat or Premature Atrial Complex or Atrial Complex, Premature or Atrial Complices, Premature or Premature Atrial Complices or Premature Atrial Contractions or Atrial Contraction, Premature or Atrial Contractions, Premature or Premature Atrial Contraction or Premature Supraventricular Beats or Premature Supraventricular Beat or Supraventricular Beat, Premature or Supraventricular Beats, Premature.

**Figure 1:**
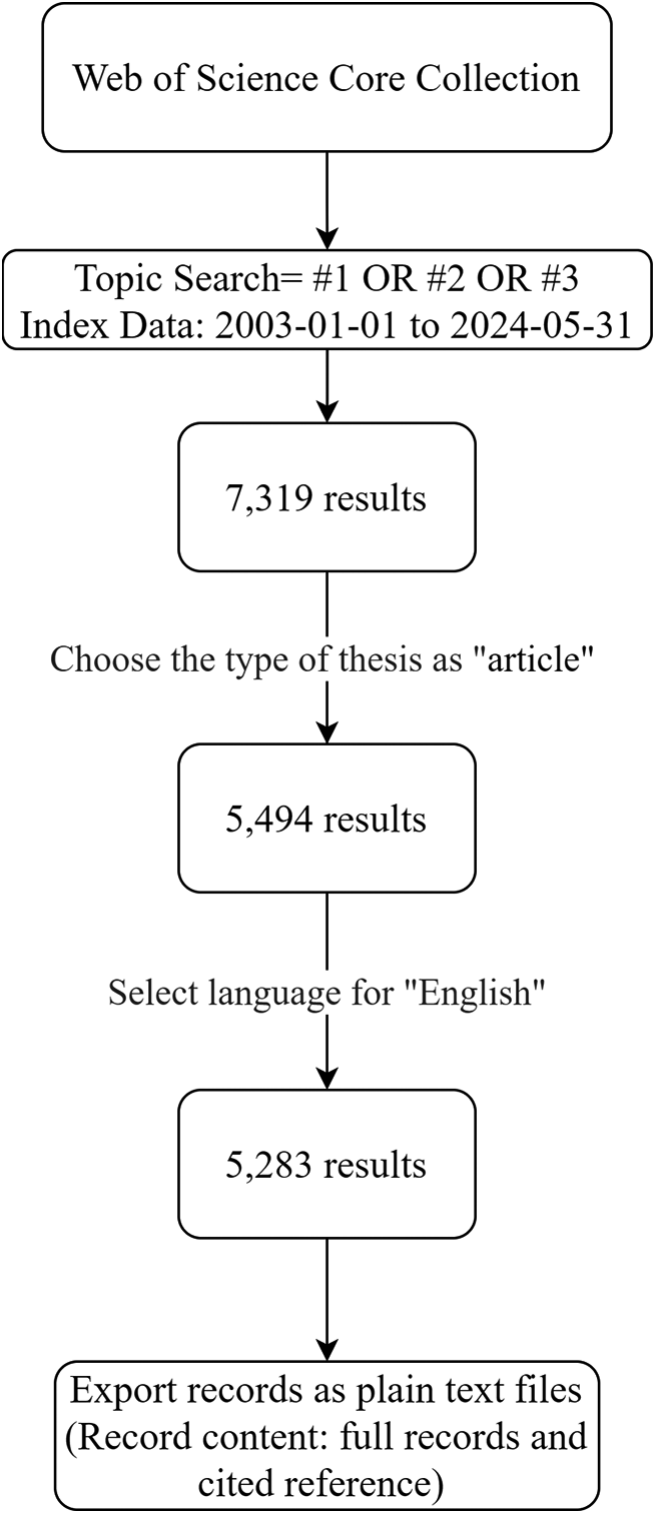
Flow chart of data source and research strategy.

The index topic was (Topic Search = #1 OR #2 OR #3). The index data range was “from 2003-01-01 to 2024-05-31”. The document type was “article” and the language was English. Data were collected on June 4, 2024. We then exported the complete records and cited references as plain text files and used VOSviewer (v.1.6.20) for co-authorship, co-citation, and co-occurrence analysis (Figure 1).

## 3 Results

### 3.1 Literature development trends

**Figure 2:**
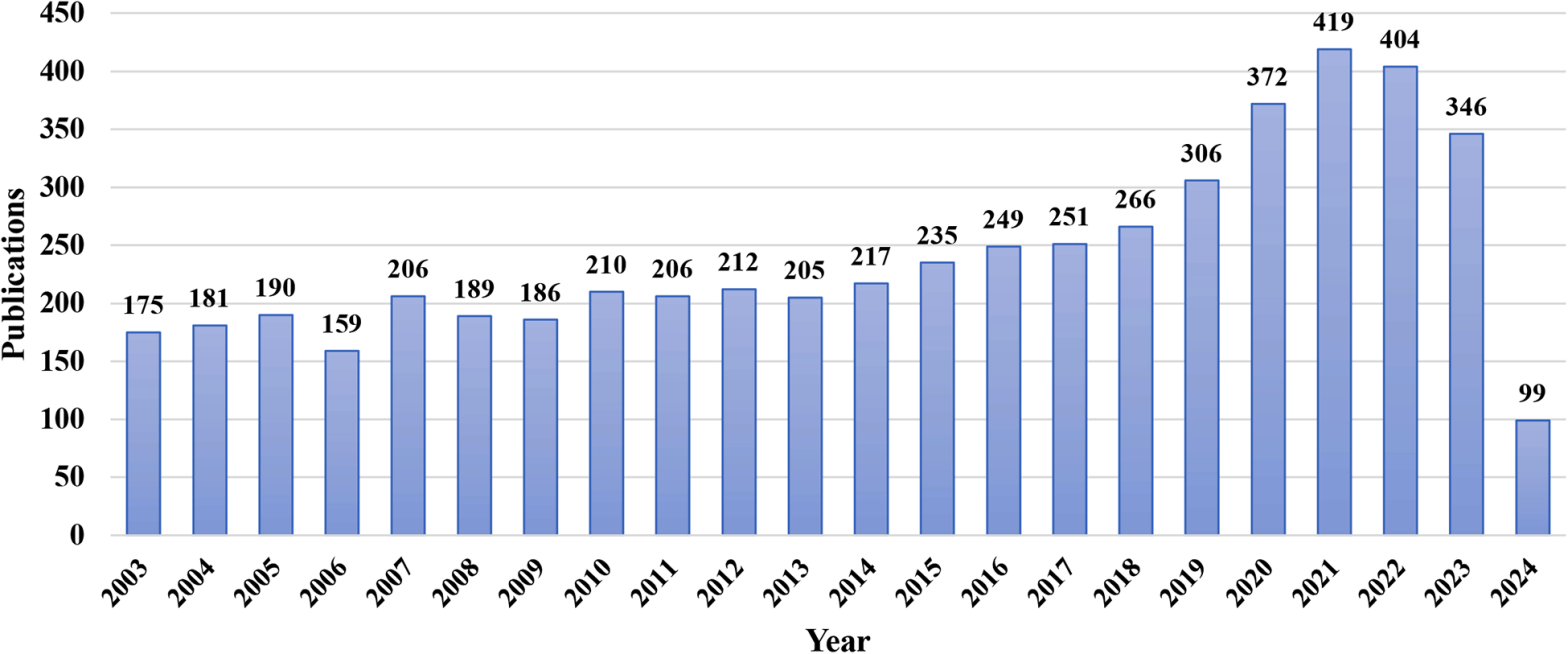
Number of annual publications from 2003 to 2024.

The number of publications over a period of time reflects the research speed and trends in this field. From 2003 to 2024, 5,283 publications on premature beats were published in WoScc. Figure 2 shows the annual publications data. From 2003 to 2014, the number of publications remained constant at 190. A total of 419 studies were published in 2021. In the recent ten years, the number of articles published has been approximately 300, indicating that premature beats have become a topic of increasing concern for scholars.

### 3.2 Co-authorship analysis

The relevance of premature beats research can be discovered by conducting a co-authorship analysis. The resulting map of key authors, organizations, and countries/regions directly displays a network of connections in premature beats research. Valuable information can guide researchers to find cooperating authors, guide organizations to know other institutions, and guide countries/regions to find development cooperation worldwide, thereby promoting high-quality development of premature beats research. In the figure created by VOSviewer, the author, institution, or countries/regions are represented by points. The larger the point, the more the articles published. A line between any two points symbolizes cooperation. The thickness of the line is known as the link strength. The thicker it is, the stronger is the collaboration.

**Figure 3:**
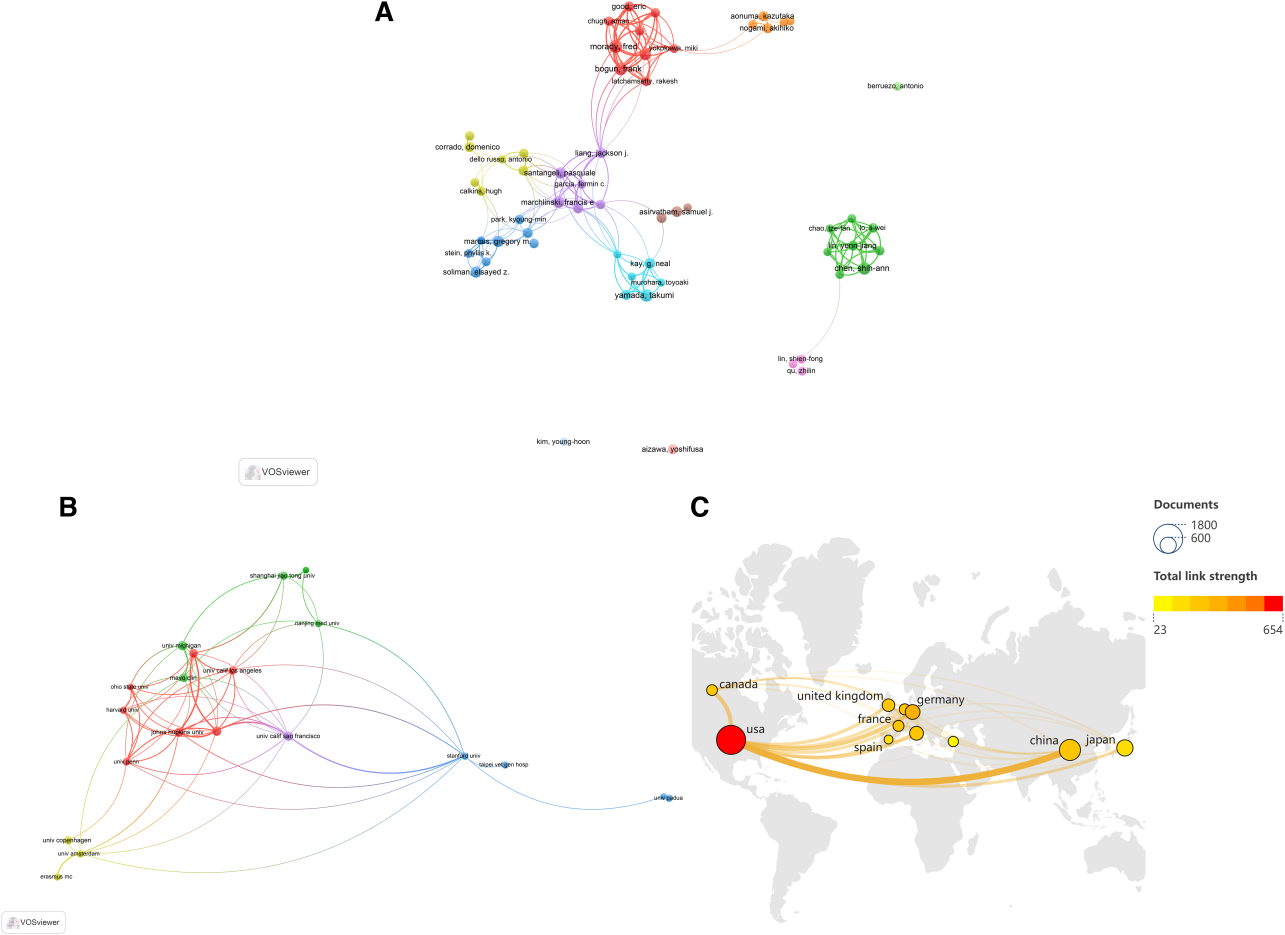
Co-authorship analysis in the premature beats research from 2003 to 2024 [(**A**) authors; (**B**) organizations; (**C**) countries/regions].

In total, 26,876 authors were involved in the field of premature beats with 5,283 publications. The VOSviewer parameters were set as follows: minimum number of documents by an author: 15; minimum number of citations by an author: 150. The results were retrieved from 26,876 authors, 58 of whom met the thresholds (Figure 3A). According to the total link strength (TLS), the top three were Fred Morady (TLS = 171), Frank Bogun (TLS = 165), and Krit Jongnarangsin (TLS = 145) (Table 1). According to VOSviewer analysis, 5,283 documents were published by 5,381 different organizations and 20 met the threshold (minimum number of documents of an organization: 36; minimum number of citations of an organization: 200) (Figure 3B). The top three were the University of Washington (TLS = 43), Johns Hopkins University (TLS = 36), and the University of Minnesota (TLS = 31) (Table 2). 94 countries/regions were involved in the publication of premature beats, and 11 met the threshold (minimum number of documents in a country: 150; minimum number of citations in a country: 200) (Figure 3C). The top three countries/regions were the United States (TLS = 654), Germany (TSL = 316), and China (TSL = 278) (Table 3).

**Table 1:** Top 10 co-authorship authors in the premature beats research from 2003 to 2024.

Rank	Author	Documents	Citations	Total link strength
1	Fred Morady	35	2,368	171
2	Frank Bogun	34	2,120	165
3	Krit Jongnarangsin	27	2,021	145
4	Shih-Ann Chen	32	463	118
5	Eric Good	20	2,195	118
6	‪Yenn-Jiang Lin	20	326	118
7	Aman Chugh	19	2,198	117
8	Hakan Oral	19	2,198	117
9	Shih-Lin Chang	19	318	116
10	Li-Wei Lo	19	325	116

**Table 2:** Top 10 co-authorship organizations in the premature beats research from 2003 to 2024.

Rank	Organization	Documents	Citations	Total link strength
1	University of Washington	65	3,015	43
2	Johns Hopkins University	67	2,429	36
3	University of Minnesota	57	1,972	31
4	University of California San Francisco	69	2,202	29
5	University of Pennsylvania	48	2,115	21
6	Stanford University	43	1,468	19
7	University of California, Los Angeles	55	2,106	18
8	The Ohio State University	37	908	15
9	Harvard University	44	1,830	13
10	Shanghai Jiao Tong University	56	612	13

**Table 3:** Top 10 co-authorship countries/regions in the premature beats research from 2003 to 2024.

Rank	Country/region	Documents	Citations	Total link strength
1	USA	1,599	52,678	654
2	Germany	413	11,408	316
3	China	842	13,527	278
4	Italy	362	9,885	270
5	Netherlands	231	6,991	260
6	United Kingdom	301	9,130	250
7	Canada	222	7,127	235
8	France	229	7,625	235
9	Spain	150	4,704	180
10	Japan	500	9,348	173

### 3.3 Co-citation analysis

Obtaining important information from cited articles enables researchers to identify mainstream research types and acquire relevant information and knowledge through co-citation analysis. The resulting map visually demonstrates the relevance of the research on premature beats. In the figure created by VOSviewer, cited references, journals, or cited authors are represented by dots. The larger the dot, the more times it has been cited, symbolizing higher academic value.

**Figure 4:**
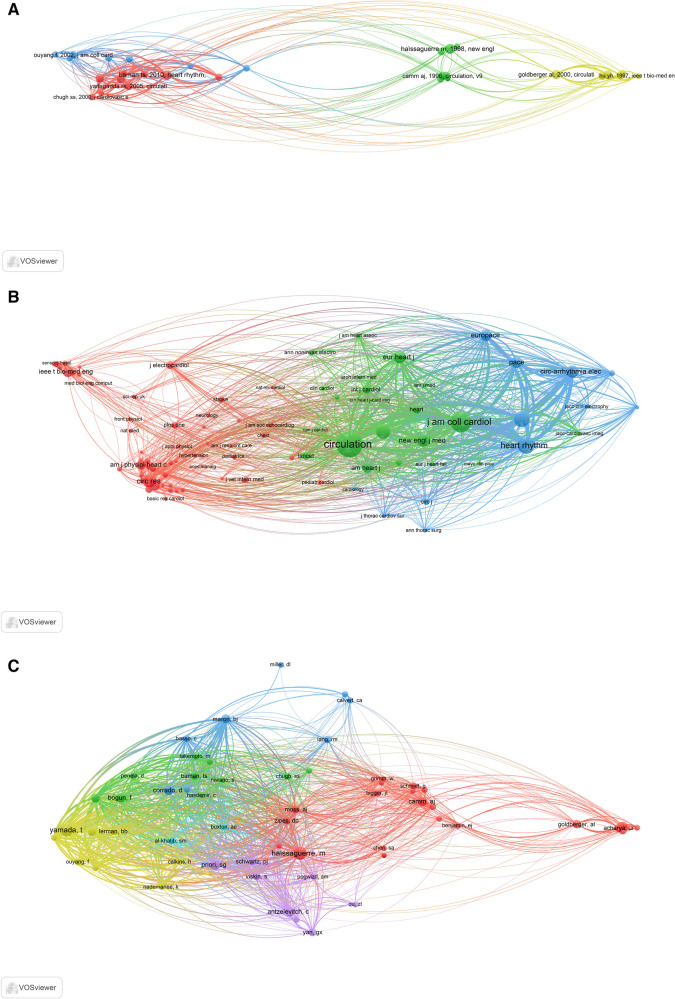
Co-citation analysis in the premature beats research from 2003 to 2024 [(**A**) cited references; (**B**) cited sources; (**C**) cited authors].

A total of 96,202 cited references were included in the field of premature beats, with 5,283 publications. The VOSviewer parameters were set as follows: minimum number of citations of a cited reference: 70. The results were retrieved from 96,202 cited references, 29 of which met the thresholds (Figure 4A). According to the number of citations, the top three were Haïssaguerre, M *et al*.*,* published in The New England Journal of Medicine in 1998, entitled “Spontaneous initiation of atrial fibrillation by ectopic beats originating in the pulmonary veins. (20)” (Citation = 212); Baman, TS et al.*,* published in Heart Rhythm in 2010 entitled “Relationship between burden of premature ventricular complexes and left ventricular function. (21)” (Citation = 194); and Goldberger, AL et al.*,* published in Circulation in 2000 entitled “PhysioBank, PhysioToolkit, and PhysioNet: components of a new research resource for complex physiologic signals. (22)” (Citation = 169) (Table 4). According to VOSviewer analysis, 96,202 cited references were published in 13,270 sources and 93 met the threshold (a minimum number of citations of a source: 200) (Figure 4B). The top three were Circulation (Citation = 13,577), Journal of the American College of Cardiology (Citation = 8,394), and Heart Rhythm (Citation = 5,823) (Table 5). Further, 96,202 cited references were written by 60,128 authors, of which 59 met the threshold (minimum number of citations per author: 100) (Figure 4C). The top three were Haïssaguerre, M (Citation = 577), Yamada, T (Citation = 548), and Priori, SG (Citation = 378) (Table 6).

**Table 4:** Top 10 cited references in the premature beats research from 2003 to 2024.

Rank	Cited reference	Citations
1	Haïssaguerre M, 1998, New Engl J Med, v339, p659, doi 10.1056/nejm199809033391003	212
2	Baman TS, 2010, Heart Rhythm, v7, p865, doi 10.1016/j.hrthm.2010.03.036	194
3	Goldberger AL, 2000, Circulation, v101, pe215, doi 10.1161/01.cir.101.23.e215	169
4	Moody GA, 2001, IEEE Eng Med Biol, v20, p45, doi 10.1109/51.932724	161
5	Bogun F, 2007, Heart Rhythm, v4, p863, doi 10.1016/j.hrthm.2007.03.003	159
6	Camm AJ, 1996, Circulation, v93, p1043	148
7	Takemoto M, 2005, J Am Coll Cardiol, v45, p1259, doi 10.1016/j.jacc.2004.12.073	147
8	Yarlagadda RK, 2005, Circulation, v112, p1092, doi 10.1161/circulationaha.105.546432	142
9	Schmidt G, 1999, Lancet, v353, p1390, doi 10.1016/s0140-6736 (98)08428-1	135
10	Pan J, 1985, IEEE T Bio-Med eng, v32, p230, doi 10.1109/tbme.1985.325532	132

**Table 5:** Top 10 cited sources in the premature beats research from 2003 to 2024.

Rank	Source	Citations
1	Circulation	13,577
2	Journal of the American College of Cardiology	8,394
3	Heart Rhythm	5,823
4	Journal of Cardiovascular Electrophysiology	4,880
5	American Journal of Cardiology	4,179
6	Circulation Research	3,411
7	The New England Journal of Medicine	3,136
8	European Heart Journal	3,003
9	Circulation: Arrhythmia and Electrophysiology	2,821
10	EP Europace	2,556

**Table 6:** Top 10 cited authors in the premature beats research from 2003 to 2024.

Rank	Author	Citations
1	Haïssaguerre, M	577
2	Yamada, T	548
3	Priori, SG	378
4	Corrado, D	315
5	Camm, AJ	296
6	Antzelevitch, C	285
7	Bogun, F	283
8	Maron, BJ	275
9	Yokokawa, M	255
10	Tada, H	239

### 3.4 Co-occurrence analysis

Co-occurrence analysis aims to identify hot topics in the field of premature beats research by analyzing the number of keywords that appear together in publications. From the generated keyword map, it is also easier to obtain advanced information in the field, which helps researchers follow the development of field research. The keywords in the figure created by VOSviewer are represented by dots. The larger the dot, the higher is its frequency of occurrence, which symbolizes the scholar's attention and current popularity.

**Figure 5:**
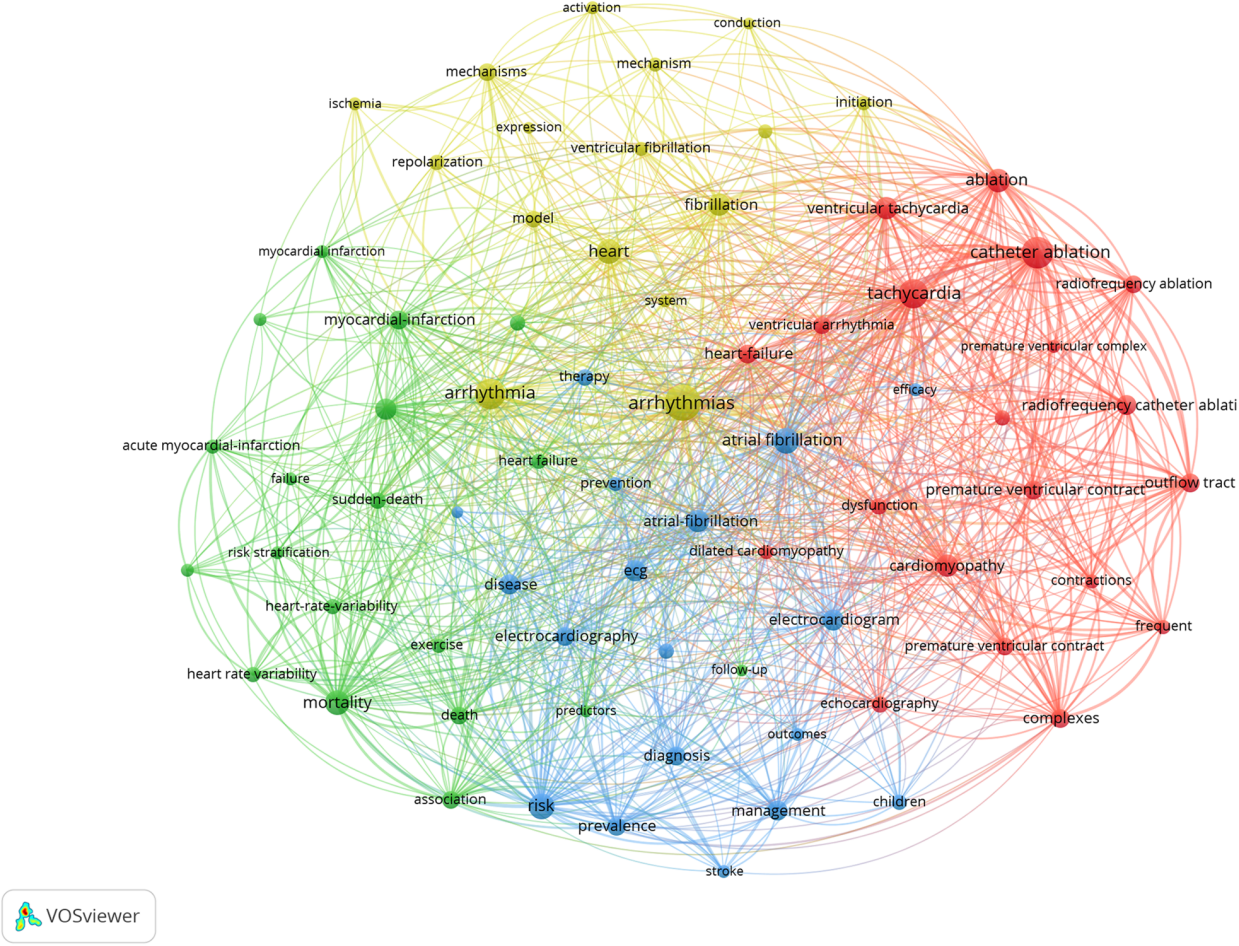
Co-occurrence analysis of keywords in the premature beats research from 2003 to 2024.

A total of 14,583 keywords were involved in the field of premature beats with 5,283 publications and 73 met the threshold (minimum number of occurrences of a keyword: 85) (Figure 5), which was mainly divided into four clusters, namely blue, yellow, green, and red (Table 7). The blue cluster primarily revolved around the risk and prevention of premature beats. The yellow cluster mainly focused on the mechanism of premature beats. The green cluster primarily revolved around disease and mortality. The red cluster primarily focused on the classification and treatment of premature beats.

**Table 7:** Cluster of keywords in the premature beats research from 2003 to 2024.

Cluster	Color	Keywords
1	Blue	Risk, atrial fibrillation, atrial-fibrillation, ecg, electrocardiogram, disease, management, diagnosis, electrocardiography, prevalence, therapy, classification, children, prevention, stroke, outcomes, efficacy, cardiac-arrhythmias
2	Yellow	Arrhythmias, arrhythmia, heart, fibrillation, mechanisms, model, repolarization, initiation, ventricular fibrillation, mechanism, electrophysiology, ischemia, activation, system, conduction, expression
3	Green	Mortality, sudden cardiac death, myocardial-infarction, death, association, sudden-death, heart failure, heart-rate-variability, heart rate variability, ventricular arrhythmias, acute myocardial-infarction, exercise, risk stratification, myocardial infarction, rate-variability, failure, ventricular-arrhythmias, follow-up, predictors
4	Red	Catheter ablation, tachycardia, ablation, cardiomyopathy, ventricular tachycardia, radiofrequency catheter ablation, heart-failure, outflow tract, premature ventricular contraction, complexes, ventricular arrhythmia, premature ventricular contractions, radiofrequency ablation, echocardiography, dysfunction, premature ventricular complexes, frequent, dilated cardiomyopathy, contractions, premature ventricular complex

## 4 Discussion

### 4.1 Research trends in premature beats

Using VOSviewer to conduct quantitative and visual analyses of research in the field of premature beats, we aimed to evaluate the development trend of premature beats research and predict its future research direction. From the dynamic changes in the total annual publication volume, we observed that research in the field of premature beats has steadily grown in recent years. With an aging global population, rapid economic development, emphasis on health, and improvements in medical care, the demand for the diagnosis and treatment of premature beats has increased significantly. It is not difficult to predict that the publications will continue to increase over the next few years.

Co-authorship analysis was used to assess and demonstrate collaborations between different authors, institutions, and countries/regions. The closeness between the authors forms a network of research relationships. The top three authors with the most TLS were all cardiac electrophysiologists and cardiologists from the University of Michigan (Table 1), mainly focusing on arrhythmia, ectopic heartbeat, and cardiac ablation, which form a very close connection (Figure 3). Co-creation can help researchers innovate and obtain new information. Paying attention to and promoting collaboration among authors will play a key role in the development of premature beats research. The nine institutions with the highest TLS among the research institutions were all in the United States (Table 2). In terms of the number of papers published and citations, the leading country was the United States (Table 3). The above findings confirm the United States's critical contributions and leading position in premature beats research, which may result from the United States' national economic conditions and high medical investment levels. This field will benefit from extensive international cooperation, which will improve the overall standard of research.

Co-citation analysis displays and illustrates the internal relationship between a large number of cited references, sources, and authors, and evaluates the impact of research on premature beats. The top three journals with the highest number of citations published high-quality papers that were recognized and cited by scholars. In Figure 4A, various frequently cited references are included. The reference with the most citations was by Haïssaguerre, M. Figure 4C shows that he also has the highest number of citations. He specializes in internal medicine, cardiology, catheter ablation, atrial fibrillation, and ablation. His research showed that the vast majority of premature atrial contractions that cause frequent paroxysmal atrial fibrillation originate from the pulmonary veins. These lesions initiate atrial fibrillation through rapid discharge and respond to local radiofrequency ablation using a catheter (20).

### 4.2 Research focus on premature beats

Hot topics and promising research directions are also presented in our study. The keywords reflected the research topic of this study. Therefore, research hotspots can be identified by analyzing the frequency of keywords in the field of premature beats. Co-occurrence analysis was used to analyze the keywords in all excerpted publications, which were mainly divided into four clusters. Based on these results, we summarized the popular topics of premature beats-related research over the past 20 years.

In the general population, the prevalence of premature ventricular contractions ranges from 3% to 20%, and increases with age, underlying heart disease, and other comorbidities (including hypertension, coronary artery disease and electrolytes) (11). Ventricular arrhythmias can manifest as asymptomatic ventricular premature syndrome or non-sustained ventricular tachycardia, symptoms of previous arrhythmias, or sustained ventricular tachycardia. Symptoms range from mild to complete hemodynamic failure (23), and studies have shown that ventricular arrhythmias are an important factor in morbidity and mortality in patients with heart failure (24). Therefore, premature ventricular beats have the greatest clinical significance and impact on the heart, which requires close attention and timely diagnosis and treatment.

Currently, three basic mechanisms underlie the occurrence of premature beats: triggered activity, automaticity, and reentry (25). Triggered activity describes premature beats and tachycardia caused by afterpotentials generated by a previous action potential (26). This is usually attributed to post-depolarization mediated by increased intracellular calcium. Clinical findings show that the typical cause of early after-depolarization is torsade de pointes secondary to long QT syndrome (referring to a group of syndromes with prolonged QT interval, ventricular arrhythmia, syncope, and sudden death on electrocardiography) (27), and the typical manifestations of late after-depolarization are digitalis intoxication or catecholaminergic polymorphic ventricular tachycardia (28). Spontaneous pacing involves ectopic pacing points that increase self-discipline; that is, the potential ectopic pacing point in different parts of the heart, when its self-discipline suddenly increases, before the basic rhythm of the excitation has not been issued or down, preemptive excitation to depolarize the atrium or ventricle, it will form premature beats (29). Reentry refers to the impulse of one part of the heart that is transmitted down the conduction path and returns to the original part to produce an impulse again (30). The classification of premature beats included the location of the origin of premature beats, number of pacing points, mechanism of occurrence, degree of early onset, and similarities and differences between the origin and dominant rhythm sites. The treatment of premature beats depends on the type of premature beats, severity of symptoms, and overall health of the patients. For most patients with premature beats, if the symptoms are not severe or there are no obvious cardiac structural abnormalities, doctors may recommend observation and lifestyle management, which includes avoiding caffeine, alcohol, and stimulants, maintaining good sleep quality, and reducing stress and anxiety. Doctors may consider using drugs to control arrhythmias during severe or frequent episodes of premature beats. Commonly used drugs include *β*-blockers, calcium antagonists, and antiarrhythmic drugs. Specific drug selection and dosage can be adjusted individually according to the patient's condition (25); for some types of premature beats, especially ventricular premature beats or atrial premature beats with severe symptoms, doctors may recommend cardiac ablation (this is an interventional procedure that burns or freezes abnormal heart tissue inside the heart through a catheter to restore normal heart rhythm) (31). In some special cases, such as cardiac structural abnormalities or the presence of other heart diseases, other treatment methods may be required, such as pacemaker implantation or surgical correction (32). Although surgery is the last-best choice for doctors, continuous monitoring and follow-up are important. The medical community's understanding of the consequences of ventricular premature beats and VPB-induced cardiomyopathy comes mainly from observational and large-scale population studies (10). In view of the current drug treatments for premature beats, the safety of long-term medication, effectiveness of drugs, and limitations of surgical treatment need to be considered. Current scholars focus on exploring the essence of premature beats and understanding the development law of premature beats.

Early detection and diagnosis are the basis for the prevention of premature beats. Ventricular arrhythmias are an important cause of morbidity and mortality and occur in many forms ranging from single premature ventricular complexes to sustained ventricular tachycardia and fibrillation. Over the past decade, the understanding of arrhythmias and their ability to diagnose them have advanced rapidly (33). With the development of new methods and tools and the publication of large-scale clinical trials, electrocardiography, Holter monitoring, event recorders, echocardiography, cardiac monitoring, and other detection techniques have been rapidly developed in hospitals or clinics for premature beats detection; these techniques are used to check or record heart-related parameters to help doctors detect and analyze the frequency, type, and related arrhythmia of premature beats (33). In the future, the continuous optimization of detection technology can quickly help doctors diagnose while reminding professional medical technicians that they need to constantly update and improve their professional knowledge. Furthermore, the results obtained through follow-up, continuous testing, and continuous summary may contribute to daily clinical practice and early intervention in the occurrence and development of premature beats as much as possible.

### 4.3 Advantages and limitations

To help new academic researchers relatively easily understand the evolution, current situation, and hotspots of premature beats research, publications on premature beats-related research were extracted from WoScc. Because all publications were evaluated based on authors, organizations, countries/regions, references, sources, and cited authors, our analysis results may provide reference values for scientists and funding agencies to explore potential partnerships and understand cutting-edge research. However, this study has some limitations. First, publications in other databases (such as PubMed, Google Scholar, and Scopus) were not considered but were only retrieved and collected from the WoScc database. Second, critical studies published in other languages may have been ignored because the analysis included only English articles. Third, although the selected publications were mainly published from 2003 to 2024, owing to the open state of the WoScc database, new research will be received continuously, and there may be some publication bias.

## 5 Conclusions

This study identified articles published between 2003 and 2024 in the field of premature beats research. We highlighted outstanding authors, organizations, countries/regions, references, sources, cited authors, and performed a keyword analysis. In summary, research on premature beats is still in its nascent stage. According to current global trends, the number of publications on premature beats research is expected to increase significantly. The United States is a dominant country in this area. Currently, all publications can be divided into four categories: “ mortality rate,” “ risk and prevention,” “ mechanism,” and “ classification and treatment.” Future research will focus on an in-depth exploration of the nature of premature beats, especially ventricular premature beats, mastering the development law of premature beats, and optimizing existing detection methods.

## Data Availability

The original contributions presented in the study are included in the article/Supplementary Material, further inquiries can be directed to the corresponding author.
